# A novel therapeutic strategy for skeletal disorders: Proof of concept of gene therapy for X-linked hypophosphatemia

**DOI:** 10.1126/sciadv.abj5018

**Published:** 2021-10-27

**Authors:** Volha V. Zhukouskaya, Louisa Jauze, Séverine Charles, Christian Leborgne, Stéphane Hilliquin, Jérémy Sadoine, Lotfi Slimani, Brigitte Baroukh, Laetitia van Wittenberghe, Natalie Danièle, Fabienne Rajas, Agnès Linglart, Federico Mingozzi, Catherine Chaussain, Claire Bardet, Giuseppe Ronzitti

**Affiliations:** 1 Genethon, 91000 Evry, France.; 2 Université Paris-Saclay, Univ Evry, Inserm, Genethon, INTEGRARE Research Unit UMR_S951, 91000 Evry, France.; 3 Université de Paris, Institut des maladies musculo-squelettiques, Laboratory Orofacial Pathologies, Imaging and Biotherapies URP2496 and FHU-DDS-Net, Dental School, and Plateforme d’Imagerie du Vivant (PIV), Montrouge, France.; 4 Paris-Saclay University, INSERM U1185, AP-HP, DMU SEA, Endocrinology and Diabetes for Children, Reference Center for Rare Diseases of the Calcium and Phosphate Metabolism, OSCAR filière, EndoRare, and BOND ERN, Bicêtre Hospital, Le Kremlin-Bicêtre, France.; 5 Institut National de la Santé et de la Recherche Médicale, U1213, Lyon F-69008, France.; 6 AP-HP, Department of Rheumatology, Cochin Hospital, Université de Paris, Paris, France.; 7 AP-HP, Reference Center for Rare Disorders of the Calcium and Phosphate Metabolism, Dental Medicine Department, Bretonneau Hospital, GHN-Université de Paris, Paris 75018, France.

## Abstract

Adeno-associated virus (AAV) vectors are a well-established gene transfer approach for rare genetic diseases. Nonetheless, some tissues, such as bone, remain refractory to AAV. X-linked hypophosphatemia (XLH) is a rare skeletal disorder associated with increased levels of fibroblast growth factor 23 (FGF23), resulting in skeletal deformities and short stature. The conventional treatment for XLH, lifelong phosphate and active vitamin D analogs supplementation, partially improves quality of life and is associated with severe long-term side effects. Recently, a monoclonal antibody against FGF23 has been approved for XLH but remains a high-cost lifelong therapy. We developed a liver-targeting AAV vector to inhibit FGF23 signaling. We showed that hepatic expression of the C-terminal tail of FGF23 corrected skeletal manifestations and osteomalacia in a XLH mouse model. Our data provide proof of concept for AAV gene transfer to treat XLH, a prototypical bone disease, further expanding the use of this modality to treat skeletal disorders.

## Teaser

A new therapeutic approach for the treatment of a skeletal disorder with AAVs bypasses bone resistance to gene transfer.

## INTRODUCTION

X-linked hypophosphatemia [XLH; OMIM (Online Mendelian Inheritance in Man) # 307800; incidence, 3.9 of 100,000 births] (*1*) is the most common form of genetic rickets associated with increased levels of circulating fibroblast growth factor 23 (FGF23). Inactivating mutations in the phosphate-regulating endopeptidase homolog X-linked (*PHEX*) gene lead to increased FGF23 secretion from osteocytes and osteoblasts, inducing renal phosphate wasting, hypophosphatemia, and impairing the endogenous production of active vitamin D [1,25(OH)_2_vitamin D] via the inhibition of 1α-hydroxylase and the activation of 24-hydroxylase (*2*). Chronic hypophosphatemia and insufficient synthesis of 1,25(OH)_2_vitamin D lead to altered bone mineralization that clinically manifests in children as rickets, i.e., severe skeletal deformities, bone pain, short stature, and severe dental abscesses. In adults, XLH is characterized by osteomalacia, pseudo-fractures, precocious and progressive osteoarthritis and enthesopathies, and poor dental health (*1*, *3*–*6*). This considerable disease burden has a considerable impact on the quality of life of affected individuals (*7*, *8*).

Standard treatment for XLH consists of multiple daily intakes of oral phosphate, to compensate the renal wasting, and active vitamin D analogs to counteract 1,25(OH)_2_vitamin D deficiency. Although commonly prescribed from infancy to the end of growth, the multiple daily doses of phosphate do not fully correct disease manifestations and are often associated with side effects and poor treatment compliance (*1*, *3*, *4*, *9*).

Recently, a new treatment based on FGF23 neutralization by a monoclonal antibody (burosumab, Crysvita, Ultragenyx) was approved for patients with XLH (*9*–*16*). Bimonthly subcutaneous injection of burosumab in children with severe XLH resulted in amelioration of rickets with improvement of impaired biochemical parameters and physical ability and reduction in patient-reported pain and functional disability (*9*, *12*, *13*). A 6- to 12-month treatment with burosumab had an impact on the main features of XLH in adult patients by accelerating healing of active fractures and pseudo-fractures and by reducing stiffness and pain (*11*, *15*, *16*). However, the high costs (*1*, *17*, *18*) and the hurdles associated with the need for repeated administrations (*1*), together with the possible insurgence of antidrug antibodies (*19*), are potential limitations of the antibody treatment. The availability of predictive preclinical models (*20*–*22*), the extensive clinical data available, and the unmet medical need make XLH an ideal target for the development of innovative strategies to treat skeletal diseases.

Adeno-associated virus (AAV) vectors have been used for in vivo gene replacement in a number of clinical trials, which support the safety and efficacy of this technology (*23*–*26*). Among the different tissues targeted with AAV vectors, the liver represents an ideal candidate due to the accessibility via the bloodstream, the efficient secretory machinery, and the protolerogenic hepatic environment (*27*). Conversely, AAV gene transfer directed to bone tissue represents an important limitation of this delivery platform. We and others previously demonstrated that liver gene therapy with AAV vectors can be exploited to secrete proteins and mediate cross-correction of enzyme deficiencies at a systemic level (*28*–*32*). On the basis of this, we hypothesized that hepatic gene transfer could be used to modulate signaling pathways in bone tissue and rescue a dominant disease.

Here, through engineering of the FGF23 sequence, we provided proof of concept of the use of the liver to secrete an interfering factor able to reduce the FGF23 pathway overactivation and rescue the bone phenotype in a murine model of XLH carrying a spontaneous deletion of *Phex*.

## RESULTS

### Design of AAV-cFGF23 vector and in vitro and in vivo efficacy evaluation

**Fig. 1.**
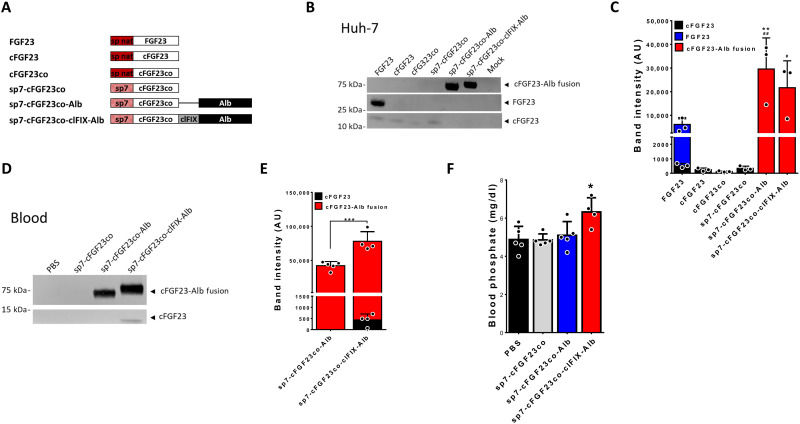
Engineering of the cFGF23 transgene for liver expression. (**A**) Description of the FGF23-derived transgenes (see Materials and Methods). (**B** and **C**) Western blot quantification of FGF23 isoform [cFGF23, FGF23, and FGF23-albumin (Alb) fusion] expression in the medium of Huh-7–transfected cells. (B) Representative Western blot. (C) Quantification of the FGF23 isoforms secreted in the medium of Huh-7 cells transfected with the indicated constructs. AU, arbitrary units. (**D** to **F**) C57BL6/J mice were injected with 1 × 10^12^ vg per mouse of AAV8 vector expressing sp7-cFGF23co, sp7-cFGF23co-Alb, or sp7-cFGF23co-clFIX-Alb under the transcriptional control of the liver-specific hAAT promoter. (D) Representative Western blot of FGF23 isoforms measured in blood 1 month after vector injection. (E) Quantification of the expression of cFGF23-Alb fusion in blood. (**F**) Blood phosphate levels measured 1 month after vector injection. Statistical analyses were performed by analysis of variance (ANOVA) in (C) (**P < 0.01 versus intact FGF23; #*P* < 0.05 and ##*P* < 0.01 versus cFGF23 measured in cells transfected with native FGF23) and in (F) (*P < 0.05) and by Student’s *t* test in (E) (***P < 0.001). All data are shown as means ± SD [*n* = 3 independent transfections in (B and C); *n* = 4 to 5 in (E and F)].

The C-terminal fragment of FGF23 (amino acids 180 to 251 of the human FGF23, cFGF23) competes with the native FGF23 for the binding to the FGF23 receptor/Klotho complex (*33*). In preclinical models, cFGF23 fusion with the Fc region of immunoglobulin G1 (IgG1) administered as recombinant protein was rapidly eliminated from the circulation and required multiple administrations per week (*34*). Given the low stability in the circulation of the cFGF23, an ideal method of delivery will require a stable infusion of the truncated factor. Hence, cFGF23 represents an ideal candidate for AAV liver gene therapy, which has a clinically proven capacity to deliver stable levels of transgenes in the circulation (*23*, *26*, *35*). To develop a stable, highly secreted form of cFGF23, we fused the human cFGF23 with the signal peptide of chymotrypsinogen B2 (sp7) (*31*) and with human albumin fused to cFGF23 either directly or through a cleavable linker derived from human coagulation factor IX (clFIX; Fig. 1A) (*36*). Those constructs were transfected in human hepatoma cells (Huh-7) in parallel with native human FGF23 and wild-type (WT) and codon-optimized versions of cFGF23 coding DNA fused with the FGF23 native signal peptide or sp7. Western blot analysis indicated that native FGF23 was efficiently produced and secreted in the conditioned medium of transfected Huh-7 cells (Fig. 1B and fig. S1A). Low levels of the cFGF23 fragment were detected despite codon optimization (co) or fusion with the sp7 signal peptide, possibly due to its poor stability in the extracellular medium (Fig. 1C). Fusion of cFGF23co with sp7 and albumin with or without the clFIX linker greatly improved the secretion in the extracellular medium in vitro (Fig. 1, B and C, and fig. S1A).

cFGF23co fusions with sp7 and albumin with or without the clFIX linker were then tested in vivo and compared to cFGF23co fused with sp7. AAV8 vectors expressing those transgenes under the transcriptional control of the liver-specific apolipoprotein E enhancer, human α1-antitrypsin promoter (hAAT) (*31*) were injected in 6-week-old C57BL/6J mice at the dose of 1 × 10^12^ vector genome (vg) per mouse (Fig. 1, D to F, and fig. S1, B and C). One month after vector injection, mice were bled to measure the expression of the three cFGF23co variants by Western blot. No bands with a size compatible with cFGF23co were detectable in mice injected with the AAV expressing cFGF23co fused with sp7 (Fig. 1D and fig. S1B). Bands corresponding to the molecular weight of cFGF23co-albumin chimeric proteins were revealed in mice injected with vectors expressing albumin fusion proteins (Fig. 1D and fig. S1B). Mice receiving the AAV vector expressing the cFGF23co-albumin fusion containing the clFIX cleavable linker showed significantly higher levels of circulating fusion protein and cleaved FGF23 when compared to its counterpart lacking clFIX (Fig. 1, D and E, and fig. S1B) despite similar transduction as demonstrated by vector genome copy number (VGCN) per diploid genome in liver (fig. S1C). Only mice injected with the AAV vector expressing the cFGF23co-albumin fusion with the clFIX cleavable linker showed increased phosphate levels compared to control animals, suggestive of decreased FGF23 signaling in the kidneys (Fig. 1F). These data support the hypothesis that the truncated FGF23 is unstable when expressed both in vitro and in vivo. They also suggest that cFGF23 fusion with albumin, despite its increased stability, has no effect on phosphatemia. In conclusion, we demonstrated that the release of cFGF23 from the albumin moiety through a cleavable linker is necessary to achieve stable circulating levels of active cFGF23. Although the exact mechanism behind the increased activity of cFGF23 after release from albumin is unknown, we can speculate that the fusion with the large protein may somehow hinder the interaction of cFGF23 peptide with its receptor.

### Liver-targeting AAV-cFGF23 is an efficient delivery platform for the treatment of XLH

**Fig. 2.**
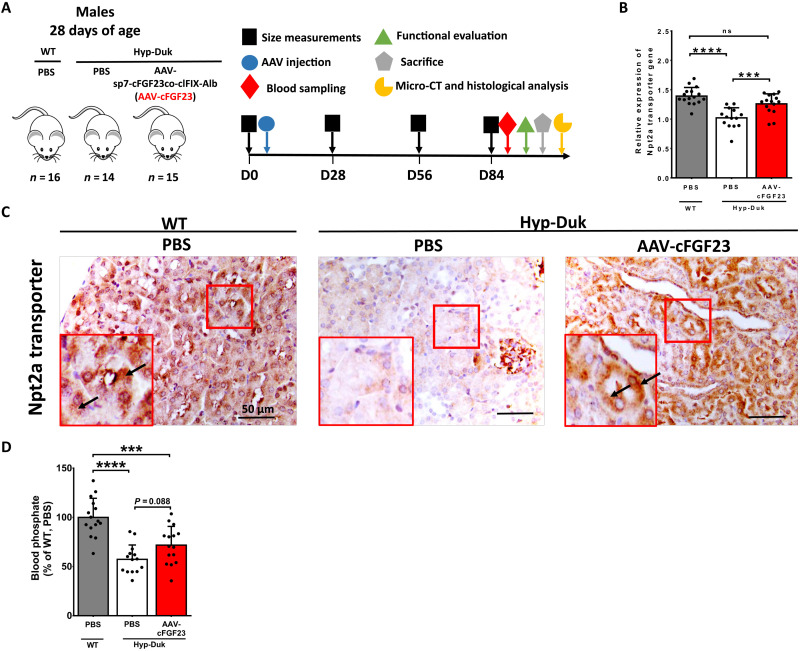
Liver expression of cFGF23 results in decreased FGF23 signaling in kidney in a mouse model of XLH. (**A** to **D**) One-month-old Hyp-Duk mice were injected with 1 × 10^12^ vg per mouse of AAV8 expressing sp7-cFGF23co-clFIX-Alb (AAV-cFGF23) and sacrificed 3 months after injection. PBS-injected WT and Hyp-Duk mice served as controls. (A) Experimental design. (B) Expression of the Npt2a transporter mRNA in kidney. (C) Expression of the Npt2a transporter (arrows) in the kidneys, obtained by immunohistochemistry. (D) Blood phosphate levels expressed as percent of WT levels measured 3 months after treatment. Statistical analyses were performed by ANOVA in (B and D) (***P < 0.001 and ****P < 0.0001; ns, not significant). All data are shown as means ± SD [*n* = 14 to 16 mice per group from three independent experiments in (B and D)]. micro-CT, micro–computed tomography.

Deletion of *Phex* leads to increased circulating FGF23, decreased sodium/phosphate cotransporter (Npt2a) expression in kidney, and the consequent phosphate wasting underlying bone disease in XLH (*2*, *21*). To verify whether liver-mediated secretion of the cFGF23-albumin fusion was able to rescue phosphate wasting in the Hyp-Duk mouse, a murine model of XLH, an AAV8 vector expressing the cFGF23co-albumin fusion with the clFIX cleavable linker (from now on called AAV-cFGF23) was injected in 4-week-old Hyp-Duk mice at the dose of 1 × 10^12^ vg per mouse (Hyp-Duk, AAV-cFGF23). Phosphate-buffered saline (PBS)–injected Hyp-Duk mice (Hyp-Duk, PBS) and WT littermates (WT, PBS) were used as controls (Fig. 2A). Under these experimental conditions, a single injection of the liver-targeted AAV-cFGF23 resulted in efficient liver targeting (fig. S1D) and restored the expression of Npt2a in kidney of AAV-injected animals, with levels of expression that were similar to those measured in WT littermates both at mRNA (Fig. 2B) and protein level (Fig. 2C). Unexpectedly, despite the complete restoration of Npt2a expression in kidney, we observed an only partial rescue of blood phosphate levels at 1 month after injection and a similar tendency at 3 months after injection of the AAV-cFGF23 in Hyp-Duk mice (Fig. 2D and fig. S1E). We then evaluated whether other Na/Pi transporters were affected in Hyp-Duk mice and rescued by the AAV-cFGF23 treatment. The levels of expression of six Na/Pi transporters were evaluated in kidney. Only type II transporters were modified in Hyp-Duk mice and rescued by the treatment (fig. S1F). Similar to Npt2a, the expression of Npt2c, another type II Na/Pi transporter, was increased in AAV-cFGF23–treated mice when compared to PBS-treated animals (fig. S1F). Npt2b, a Na/Pi transporter only residually expressed in kidney (*37*, *38*), was increased in Hyp-Duk mice and rescued by the AAV-cFGF23 treatment (fig. S1F). These results support the hypothesis of a different role for the three type II Na/Pi transporters in the pathogenesis of XLH. Regardless of their role, their levels in kidney are normalized by the treatment.

In XLH, inappropriately normal levels of circulating 1,25(OH)_2_vitamin D were reported. One potential secondary effect of the interference with FGF23, which inhibits the synthesis of 1,25(OH)_2_vitamin D in physiological condition, could be the overactivation of its synthesis. Proof of the potential risk of increasing the synthesis of calcitriol was obtained in Fgf23 knockout mice that have high blood levels of active vitamin D and ectopic calcifications. These calcifications disappeared when these animals were crossed with mice knockout for 1α-hydroxylase (*39*), the main enzyme of 1,25(OH)_2_vitamin D synthesis, thus suggesting that high levels of calcitriol may be associated with the formation of ectopic calcifications. Importantly, the inhibition of the FGF23 signaling through the administration of cFGF23 as a peptide in XLH mice was associated with increased serum phosphate and normal levels of 1,25(OH)_2_vitamin D (*34*).

To assess the safety profile of our therapeutic strategy, we evaluated the levels of expression of 1α-hydroxylase and 24-hydroxylase, key enzymes in 1,25(OH)_2_vitamin D synthesis and inactivation, respectively. Three months after AAV-cFGF23 vector injection, the levels of expression of 1α-hydroxylase and 24-hydroxylase were measured in kidney, the primary site of production of these molecules. Both enzymes were increased in Hyp-Duk untreated mice and did not change after 3 months of AAV-cFGF23 treatment (fig. S2, A and B). In line with these results, blood circulating levels of 1,25(OH)_2_vitamin D were unchanged across treatment groups (fig. S2C). These findings suggest that the inappropriately normal levels of 1,25(OH)_2_vitamin D in hypophosphatemic Hyp-Duk mice are possibly the consequence of a balance between increased synthesis and degradation of active vitamin D. They also indicate that AAV-cFGF23 has no effect on the vitamin D metabolism in kidney.

Despite unchanged levels of active vitamin D (fig. S2C), calcium levels in blood measured at 1 and 3 months after injection were significantly decreased in untreated Hyp-Duk mice and were restored in AAV-cFGF23–treated Hyp-Duk mice to levels similar to those measured in WT animals (fig. S2, D and E). No evidence of kidney calcification was shown by Von Kossa staining in animals of the three groups (fig. S2F).

### AAV-cFGF23 treatment rescues bone microarchitecture, mineralization, and growth plate morphology

**Fig. 3.**
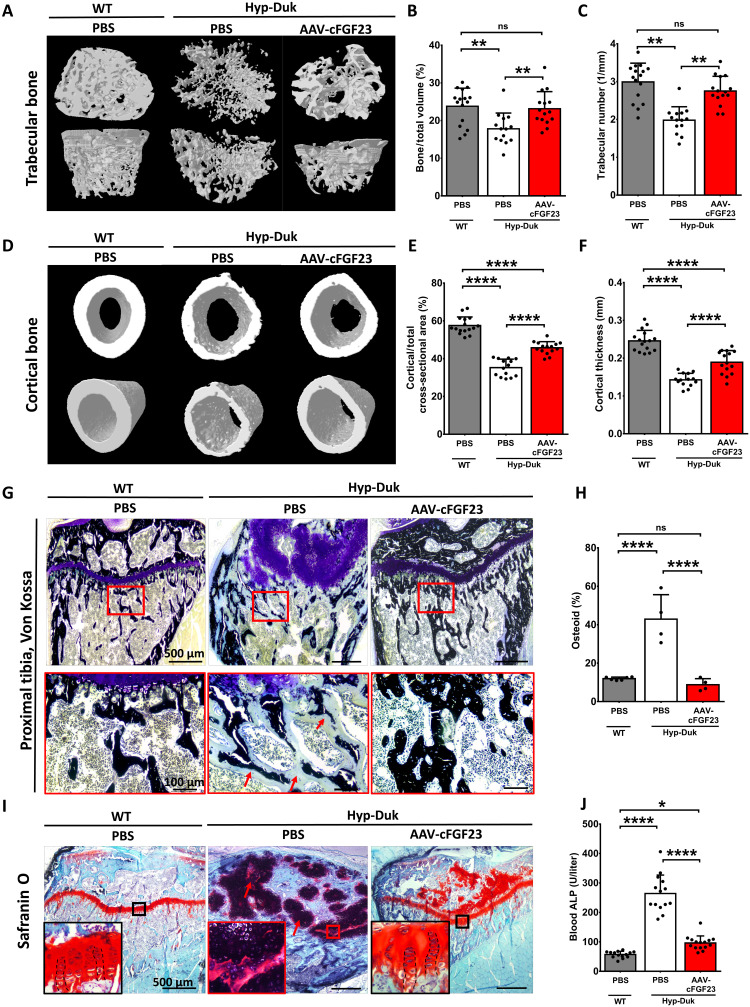
AAV-cFGF23 treatment rescues bone microarchitecture, mineralization, and growth plate morphology. One-month-old Hyp-Duk and WT mice were treated as described in Fig. 2. (**A**) Micro-CT images of the trabecular bone from femur. (**B**) Bone–to–total volume ratio in the trabecular bone, expressed in percent. (**C**) Trabecular number, expressed as trabeculae/mm. (**D**) Micro-CT images of the cortical bone from femur. (**E**) Cortical area to total cross-section area ratio, expressed in percent. (**F**) Thickness of the cortical bone wall, expressed in millimeter. (**G**) Von Kossa staining performed on the proximal tibia. Correctly mineralized areas are stained dark. Red arrows indicate the enlarged areas of nonmineralized collagenous matrix (osteoid, in light blue). (**H**) Quantification of osteoid in histological series of Von Kossa staining, expressed in percent of total bone area. (**I**) Safranin O staining of the proximal tibia, showing the structure of proximal metaphyseal growth plate. Red arrows indicate hypertrophic chondrocytes. (**J**) Blood levels of ALP measured 3 months after the injection of the AAV-cFGF23 or PBS. Statistical analyses were performed by ANOVA (*P < 0.05, **P < 0.01, and ****P < 0.0001). All data are shown as means ± SD [*n* = 14 to 16 mice per group from three independent experiments in (B, C, E, F, and J); *n* = 4 to 6 mice per group in (H)].

Three months after vector injection, micro–computed tomography (micro-CT) analysis of the trabecular bone revealed complete restoration of the bone structure (Fig. 3A). In particular, AAV treatment in Hyp-Duk mice completely rescued three important parameters (i) the trabecular structure, namely, the bone volume–to–total volume ratio (Fig. 3B), (ii) the number of trabeculae per millimeter (Fig. 3C), and (iii) the trabecular separation (fig. S3A), to values similar to those measured in WT animals. Significant rescue of the trabecular pattern in addition to substantial rescue of the trabecular thickness were also indicative of the improvement in bone microarchitecture (fig. S3, B and C). AAV-cFGF23 treatment also improved the structure of the cortical bone (Fig. 3D) such as the ratio cortical to total cross-sectional area and the cortical thickness (Fig. 3, E and F). Improved bone structure was further confirmed by histological analyses. Von Kossa staining of proximal tibia showed increased bone mineralization in the ossification zone in AAV-treated Hyp-Duk mice (Fig. 3G). The enlarged osteoid observed in untreated Hyp-Duk mice was substituted by mineralized bone after treatment with AAV-cFGF23, with a level similar to that measured in WT animals (Fig. 3, G and H). Consequently, AAV-treated Hyp-Duk mice showed fainter alkaline phosphatase (ALP) staining, a marker of osteogenic cell activity, compared to untreated Hyp-Duk mice, which displayed areas of strong ALP activity at the periphery of the accumulated osteoid (fig. S3, D and E). In addition, tartrate-resistant acid phosphatase (TRAP) staining, a marker of osteoclasts, was observed along mineralized trabeculae in AAV-treated Hyp-Duk mice with a distribution similar to WT mice (fig. S3F).

### AAV-cFGF23 increases bone growth and rescues sacroiliac joint structure

Consistently with the amelioration of bone microarchitecture, we observed substantial improvement of the growth plate structure, responsible for bone elongation, as evidenced by Safranin O staining (Fig. 3I). In untreated Hyp-Duk mice, the columnar structure of chondrocytes in growth plates was severely disorganized, which resulted in the abnormal thickening of the growth plate structure. AAV-cFGF23 treatment restored the columnar organization of the chondrocytes and decreased the total thickness of the growth plate (Fig. 3I). Consistently, blood levels of total ALP, a good marker of skeletal remodeling (*40*, *41*), was significantly decreased in AAV-cFGF23–treated Hyp-Duk mice when compared with PBS-treated Hyp-Duk counterparts (Fig. 3J). Together, these results indicate that AAV-cFGF23 treatment in 1-month-old animals normalized bone mineralization and growth plate abnormalities.

**Fig. 4.**
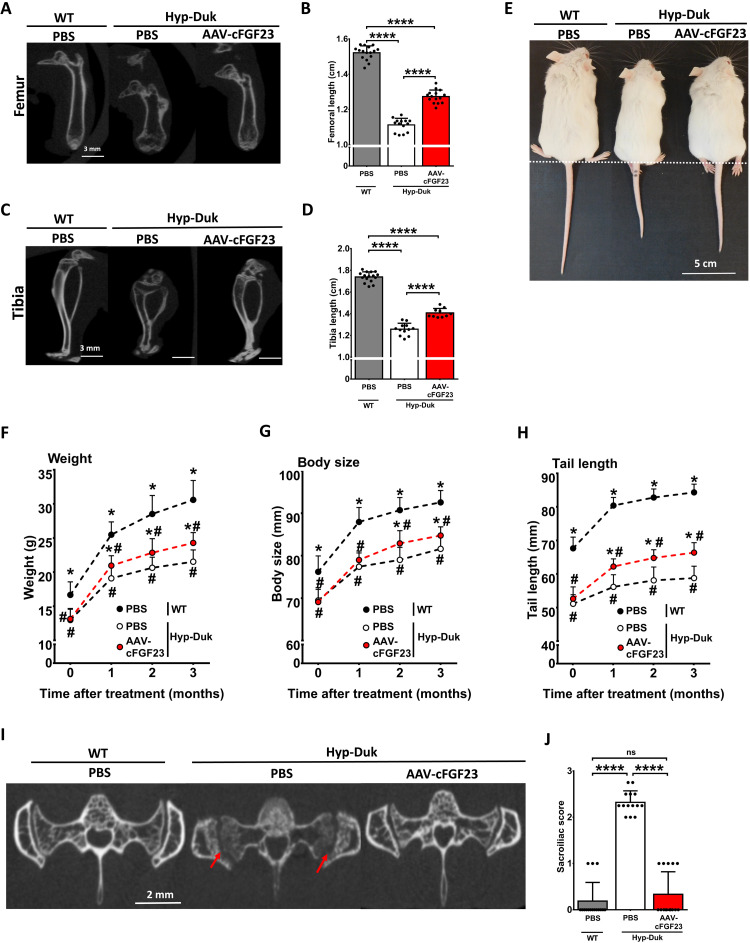
AAV-cFGF23 increases bone growth at whole body level and rescues sacroiliac joint structure. One-month-old Hyp-Duk and WT mice were treated as described in Fig. 2. Mice were monthly measured starting from day zero to evaluate the morphometric parameters. (**A** to **D**) Micro-CT images of femur (A) and tibia (C) acquired 3 months after injection. Femur (B) and tibia (D) length as measured on the micro-CT images. (**E**) Picture of PBS-treated WT and Hyp-Duk mice treated with PBS or AAV-cFGF23 taken 3 months after the injection. The dashed line represents the beginning of the tail. Photo credit: Severine Charles, Genethon. (**F** to **H**) Morphometric parameters measured before and 1, 2, and 3 months after AAV-cFGF23 or PBS treatment in WT and Hyp-Duk animals. (F) Body weight. (G) Body size. (H) Tail length. (**I**) Micro-CT images of sacroiliac joint 3 months after the injection. Multiple erosions and irregular cortical board are indicated by red arrows. (**J**) Scoring of the sacroiliac joint degeneration based on micro-CT imaging 3 months after AAV-cFGF23 or PBS injections. Statistical analyses were performed by ANOVA in (B, D, and J) (****P < 0.0001) and two-way ANOVA in (F to H) (#*P* < 0.05 versus WT, PBS; *P < 0.05 versus Hyp-Duk, PBS). All data are shown as means ± SD (*n* = 14 to 16 mice per group from three independent experiments).

We next evaluated the effect of liver gene therapy on the restoration of long bone growth. AAV-cFGF23 injection promoted the elongation of femur and tibia in treated mice together with a general amelioration of the bone structure and a reduction of the distorted epiphysis and diaphysis typically observed in Hyp-Duk mice (Fig. 4, A to D). Phenotypically, AAV-treated Hyp-Duk animals were larger (Fig. 4E) with significantly improved weight overtime (Fig. 4F). Consistent with the elongation observed in long bones (Fig. 4, A to D), we observed a partial rescue of body and tail length in Hyp-Duk mice after AAV-cFGF23 treatment, suggestive of a general positive effect on the longitudinal bone elongation (Fig. 4, G and H).

Osteoarticular rachitic manifestations are typical in adult patients with XLH (*7*) and were also reported in hypophosphatemic mice (*6*). We then evaluated the effect of the treatment on sacroiliac arthritis. As expected, untreated Hyp-Duk mice showed multiple erosions and an irregular aspect of the cortical board by micro-CT with higher sacroiliac score compared to WT animals (Fig. 4, I and J, and table S1). In AAV-treated Hyp-Duk mice, the treatment rescued signs of sacroiliac arthritis both in terms of morphology and sacroiliac score (Fig. 4, I and J, and table S1). In addition, total body micro-CT scan showed that AAV-treated Hyp-Duk presented with skeleton features similar to WT mice with a restoration of vertebral body hypertrophy (fig. S4, A and B). Consistently with the general improvement in bone architecture, a significant reduction in the number of falls in four-limb hanging test was seen in AAV-treated Hyp-Duk mice 3 months after vector injection to levels similar to those measured in WT animals (fig. S4C).

## DISCUSSION

XLH is due to inactivating mutations in *PHEX* associated with increased secretion of FGF23 by osteocytes and osteoblasts. Correction of the genetic defect through *PHEX* replacement directed to those cells may prove difficult regarding their refractory to AAV vectors. Recently, several groups have been focused on the engineering of the AAV capsid to increase bone targeting, resulting also in increased risks to transduce nonbone tissues (*42*, *43*). Gene transfer strategies for skeletal diseases remain challenging nowadays.

Here, as an alternative to direct targeting of the bone cells, we propose an innovative strategy to bypass bone targeting by using liver as a biofactory to secrete a negative regulator of the FGF23 signaling pathway and reduce phosphate wasting, the main cause of poor bone mineralization in XLH. Through optimization of the delivery platform, we demonstrated that treatment with AAV-cFGF23 inhibited FGF23 signaling in kidney, resulting in increased expression of the Npt2a transporter and complete rescue of bone mineralization and osteomalacia in a mouse model of XLH. In addition, different from the approved anti-FGF23 monoclonal antibody treatment, in patients with XLH (*10*), AAV-cFGF23 did not affect blood levels of active vitamin D or the expression of key enzymes involved in its synthesis and degradation and did not trigger calcium deposition in the kidneys of Hyp-Duk mice. Moreover, AAV-cFGF23 completely rescued circulating calcium levels in animals. Future studies will help elucidate the molecular mechanism behind the selective effect of cFGF23 on circulating calcium levels, which, in turn, may constitute an additional safety advantage of gene therapy as a long-term treatment for XLH.

Treatment with AAV-cFGF23 in 1-month-old mice resulted in complete rescue of bone structure but partially corrected bone length. This apparent discrepancy is possibly related to the fact that we treated mice at an age where the growth plate, the developmental center for endochondral ossification involved in linear growth, is almost completely mature (*44*). Although this age was selected because gene transfer to the liver results in stable expression of the transgene (*45*), we cannot exclude that AAV treatment at a younger age (i.e., 2 weeks) may still provide sufficient transgene expression and complete bone length restoration. Another option may be the repeated administration of AAV vectors starting from the postnatal period. The formation of anti-AAV neutralizing antibodies represents a limitation to this approach. We recently proposed the use of immunomodulation with rapamycin-containing nanoparticles (*46*) or by the IgG-degrading enzyme IdeS (*47*) as strategies to enable vector readministration. These findings are changing the paradigm of AAV gene therapy as a one-time treatment and, if successfully translated to the clinic, they may allow for the correction of XLH manifestations in young pediatric subjects.

One potential limitation of our study is the lack of data supporting efficacy in older mice. Future studies will help in evaluating whether the administration of AAV-cFGF23 can have beneficial effects at an advanced stage of the disease. This will provide crucial information on the optimal window of time in which gene therapy can be clinically relevant while generating insights into the disease pathophysiology and reversibility.

Although we cannot exclude that the partial rescue of phosphate levels in blood is due to phosphate reallocation to bone metabolism, one hypothesis is that it could be derived from the increased expression of Npt2b in Hyp-Duk mice kidney. Although the role of this Na/Pi cotransporter in the pathophysiology of XLH is still unknown, further investigations are ongoing to pinpoint its role in FGF23 signaling in kidney and in other organs where Npt2b transporter plays a central role in phosphate homeostasis, for example, the intestine (*48*).

In conclusion, our results provide proof of concept of efficacy of liver-targeted AAV gene therapy in XLH mice. They also support the use of the liver as a platform to secrete factors able to modulate pathways in tissues refractory to AAV gene therapy, such as bone or kidney, further expanding the therapeutic potential of the approach.

## MATERIALS AND METHODS

### Cloning of the FGF23 constructs

The different FGF23 constructs were cloned into an expression cassette optimized for liver expression, including an enhancer derived from apolipoprotein E gene, hAAT, the human hemoglobin subunit β (HBB2)–derived synthetic intron, and the HBB2 polyadenylation signal (*49*). Different variants of FGF23 were generated on the basis of the coding sequence of human FGF23. Full-length, native FGF23 (NM_020638) was expressed including its natural signal peptide (nucleotides 1 to 72 of the FGF23 sequence). cFGF23 constructs corresponded to amino acids 180 to 251 of the human FGF23 protein (NP_065689) fused either with the native signal peptide (sp-nat, amino acids 1 to 24 of NP_065689) or with the chymotrypsinogen B2 signal peptide (sp7, amino acids 1 to 18 of NP_00102037) (*31*). Codon-optimized (co) cFGF23 fused with the sp7 signal peptide was expressed in fusion with human serum albumin without its signal peptide (albumin, amino acids 19 to 609 of NP_000468). A cleavable linker derived from human clFIX (amino acids 182 to 200 of CCA61112) was included in one of the sequences.

### AAV vectors production

AAV vectors used in this study were produced using a slight modification of the adenovirus-free transient transfection methods and purified as described earlier. Briefly, adherent human embryonic kidney cells (HEK293) grown in roller bottles were transfected with the three plasmids containing the adenovirus helper proteins, the AAV Rep and Cap genes, and the inverted terminal repeat–flanked transgene expression cassette. After 72 hours of transfection, cells were harvested, lysed by sonication, and treated with Benzonase (Merck-Millipore, Darmstadt, Germany). Vectors were then purified using two successive ultracentrifugation rounds in cesium chloride density gradients. Full capsids were collected; the final product was formulated in sterile PBS containing 0.001% of Pluronic (Sigma-Aldrich, Saint Louis, MO) and stored at −80°C. Titers of AAV vector stocks were determined by SDS–polyacrylamide gel electrophoresis (SDS-PAGE) followed by SYPRO Ruby protein gel stain and band densitometry.

### In vitro study

Human hepatocellular carcinoma cells (Huh-7) were transfected by Lipofectamine 2000 (Thermo Fisher Scientific, Waltham, MA) with 2 μg of the FGF23 constructs. Two days after transfection, the medium was harvested and the levels of FGF23 were analyzed by Western blot. The experiment was performed three times.

### Western blot

Huh-7 medium after transfection and serum from mice injected with the different AAV vectors were loaded onto a 4 to 12% gradient polyacrylamide gel to perform SDS-PAGE. After transfer on nitrocellulose, the membrane was blocked and incubated with an anti-FGF23 antibody (R&D System, Minneapolis, MN). The membrane was incubated with the appropriate secondary antibody (LI-COR Biosciences, Lincoln, NE) and then visualized by Odyssey imaging system and the Image Studio Lite (v5.2) software (LI-COR Biosciences).

### In vivo study

Mouse studies were performed according to the French and European legislation on animal care and experimentation (2010/63/EU) and approved by the local Institutional Ethical Board (protocol number 2019-004). Six-week-old C57BL6/J mice were purchased from Charles River Laboratory (Ecully, France) and administered with AAV vectors at 1 × 10^12^ vg per mouse intravenously via the tail vein. One month after vector injection, the levels of FGF23 isoforms were measured in circulation by Western blot.

In vivo studies were also performed in Hyp-Duk mice obtained from the Jackson Laboratory (Bar Harbor, ME). The in vivo study in Hyp-Duk mice consisted of three independent experiments. Each experiment contained three groups of mice: PBS-treated WT mice (WT, PBS), PBS-treated *HypDuk*^−/*0*^ mice (Hyp-Duk, PBS), and AAV-cFGF23–treated *HypDuk*^−/*0*^ (Hyp-Duk, AAV-cFGF23). The final number of mice used in this study was the following: 16 WT, PBS; 14 Hyp-Duk, PBS; and 15 Hyp-Duk, AAV-cFGF23.

All mice were housed in standard conditions of temperature (23° ± 2°C) in a light-controlled environment, with unlimited access to water and standard pelleted food. Given the small size of the animals, in some cases, mice had gel food supplementation (DietGel Recovery, Clear H_2_O, Portland, ME) from birth until 7 days after injection. DietGel Recovery composition indicated by the supplier is as follows: purified water, corn syrup, vegetable oil, hydrocolloids, wheat protein, electrolyte mix, food acid, and mineral mix. No phenotype differences were observed between groups with and without the supplementation; however, the number of pups surviving among the litter was markedly increased by adding the gel. Mouse genotype was determined by quantitative polymerase chain reaction (qPCR) using primers for *Phex* gene, on exon 14: forward, 5′-GATCAAATTTTCAGAATCGGACTAC-3′ and reverse, 5′-CCAGAAGAAATCAGACTGTGC-3′. WT and *HypDuk*^−/*0*^ littermate males were used for this study.

The treatment consisted of intravenous injection of AAV-cFGF23 administered at the dose of 1 × 10^12^ vg per mouse at 4 weeks of age. Mice were sacrificed after 3 months when they were at 4 months of age.

#### Growth parameters

Morphometric parameters, i.e., body weight, total body, and tail length, were measured on mice anesthetized with isoflurane before the treatment and thereafter, every month until sacrifice. Precision scales and a graduated ruler were used for weight and length measurements, respectively. Body length was considered from the muzzle to the beginning of the tail and tail length from the beginning of the tail to its end.

#### Blood parameters

Blood sample collection was performed from mice anesthetized with isoflurane before injection and 1 and 3 months later. At the latter times of follow-up, blood parameters were measured: phosphate, calcitriol [1,25(OH)_2_vitamin D], calcium (Ca^2+^), and ALP.

Phosphatemia and ALP were measured in sera by a colorimetric reaction performed with the FUJI DRI-CHEM NX500 analyzer (FUJIFILM Corporation, Tokyo, Japan). 1,25(OH)_2_vitamin D blood levels were assessed on sera by radioimmunoassay (Immunodiagnostic Systems, Boldon, UK).

#### Functional evaluation

Four-limb hanging test was performed (after 3 months of treatment) by hanging the mouse on a grid that was then flipped over. The number of falls over a period of 3 min was recorded. The average number of falls per minute was reported for each animal.

### Ex vivo study

#### mRNA expression

RNA was extracted from 50 to 100 mg of kidney tissue, using TRIzol (Thermo Fisher Scientific) according to the manufacturer’s instructions. cDNA was obtained by real-time qPCR (RT-qPCR) performed using RevertAid H Minus First Strand cDNA Synthesis Kit (Thermo Fisher Scientific).

To quantify relative gene expression, RT-PCR was performed using TaqMan gene expression assays (Thermo Fisher Scientific) on a LightCycler 480 Real-Time PCR System (Roche, Basel, Switzerland). The following TaqMan gene expression assays were used: 1α-hydroxylase (*Cyp27b1* gene, Mm01165918-g1), 24-hydroxylase (*Cyp24a1* gene, Mm00487244-m1), Npt-1 (*Slc17a1* gene, Mm00436577-m1), Npt2a (Slc34a1 gene, Mm00441450-m1), Npt2b (Slc34a2 gene, Mm01215846-m1), Npt2c (Slc34a3 gene, Mm00551746-m1), PiT1 (Slc20a1, Mm00489378-m1), PiT2 (Slc20a2, Mm00660203-m1), and glyceraldehyde 3-phosphate dehydrogenase (GAPDH; Mm99999915-g1) used as house-keeping gene. Each sample was tested in duplicate into two repeated measures. *C*_t_ values were averaged, and the amount of mRNA relative to GAPDH was calculated using the ΔΔ*C*_t_ method according to the formula RQ = 2^−ΔΔ*C*t^.

#### Vector genome copy number

VGCNs were measured by RT-qPCR. Total DNA was extracted from 100 mg of liver tissue, using MagNA Pure 96 DNA (Roche) according to the manufacturer’s instructions. The RT-qPCR was performed in LightCycler 480 Real-Time PCR System (Roche) using SYBR Green mix (Thermo Fisher Scientific). Specific primers were as follows: forward, 5′-GGCGGGCGACTCAGATC-3′ and reverse, 5′-GGGAGGCTGCTGGTGAATATT-3′.

#### Micro–x-ray CT analysis

All the mice were scanned after sacrifice using a high-resolution x-ray micro-CT device (Quantum FX Caliper, Life Sciences, Perkin Elmer, Waltham, MA) hosted by the PIV (Plateforme Imageries du Vivant) Platform, URP2496, Montrouge, France. Acquisition and analysis were performed following published guidelines (*50*). Briefly, standard acquisition settings were applied (90 kV, 160 mA), and scans were performed with a field of view alternatively focused on the sacroiliac joint (120 s, 59-μm^3^ voxel size), the hip (180 s, 59-μm^3^ voxel size), and the spine (120-s scan time, 40-μm^3^ voxel size) and covering the full hip and tibia and the full body (26 s, 236-μm^3^ voxel size). Micro-CT datasets were analyzed using the inbuilt multiplanar reconstruction tool of OsiriX software 5.8 (Pixmeo, Switzerland) to achieve series of images aligned over time for each anatomical region of each animal.

Sagittal images of femur and tibia were used to take the measures of femur and tibia length. Images of full total body, of the dorsal spine, and of the sacroiliac joints were reconstructed. Sacroiliac osteoarthritis was defined as the presence of demineralization of iliac bone, erosions, and irregular board of sacroiliac joint. Erosion of the sacroiliac joints was assessed as described previously (*6*). The reader was blind to the status of the mouse. A semiquantitative score was established, ranging from 0 (normal) to 3 (most severe feature assessed) for sacroiliac erosions (table S2).

Trabecular and cortical bones were analyzed at distal metaphysis and mid-diaphysis of femur, respectively (180-s scan time, 10-μm voxel size). For trabecular analysis, first, image stacks were oriented in the same direction using DataViewer (SkyScan, release 1.5.2.4; Kontich, Belgium) to determine the region of interest (ROI). Second, quantification of the distal metaphysis bone microarchitecture below the growth plate was performed using CT scan Analyser software (SkyScan, release 1.13.5.1). ROI was drawn in the transverse sections along the cortical zone. Batch processing was applied on our data including a median filter (with radius 1) for noise reduction, and adaptive thresholding was performed with a radius of 1. Following segmentation, batch processing calculated three-dimensional (3D) parameters of the binary image (*50*): bone volume/total volume, trabecular number, trabecular separation, trabecular thickness, and trabecular pattern factor (a marker of trabeculae connectedness). For cortical bone analysis, image stacks were only oriented in the same direction and batch processing was applied on our data, including a median filter (with radius 1) for noise reduction, and adaptive thresholding was performed with a radius of 1. Following segmentation, batch processing calculated 3D parameters of the binary image (*50*): ratio cortical/total cross-sectional area and cortical thickness. Since the femur length of Hyp-Duk mice is significantly shorter than in WT mice, the ROIs were adjusted according to the femur size (*50*). The average femur length of WT mouse in our study was equal to 15.3 mm. Two hundred slices in each ROI (distal metaphysis of femur for trabecular bone and mid-diaphysis of femur for cortical bone) taken in analyses correspond to 2 mm, which is equal to 13.1% of total femur length (2 mm/15.3 mm × 100 = 13.1%). Consequently, the ROIs for each femur were adjusted according to this calculation to obtain the 13.1% of total femur length. 3D visualization was performed with software CTVol (SkyScan, release 2.2.3.0).

#### Murine bone tissue preparation

After sacrifice, bones were fixed overnight at 4°C in 70% ethanol solution and dehydrated in a graded ethanol series. Undecalcified samples were embedded in methyl methacrylate (Merck, Rahway, NJ). Serial sections, 5 μm thick, were cut on a microtome (Polycut E microtome, Leica, Wetzlar, Germany). Series of consecutive sections were stained with Von Kossa (5% silver nitrate solution; Sigma-Aldrich), counterstained with toluidine blue (pH 3.8), and stained with Safranin O Lillie’s Trichrome (Sigma-Aldrich).

#### Enzyme histochemistry

TRAP was used to evaluate osteoclasts by using 2.5 mM naphthol AS-TR phosphate (Sigma-Aldrich), 0.36 M *N*-*N*-dimethyl-formamide (Sigma-Aldrich), and 4 mM salt in pH 5.2 acetate buffer. Nonosteoclastic acid phosphatase activity was inhibited with 100 mM l(+)-tartaric acid (Sigma-Aldrich) added to the substrate solution.

ALP was used to reveal the layer of osteogenic cells (pre-osteo/cementoblasts and osteo/cementoblasts) by incubating the sections with naphthol-ASTR-phosphate (Sigma-Aldrich) and diazonium Fast Blue RR salt (Sigma-Aldrich) for 30 min at 37°C (pH 9) in the presence of MgCl_2_.

#### Immunohistochemistry for Npt2a transporter expression

Kidney sections embedded in paraffin were deparaffinized in toluene. After rehydration in a graded ethanol series to pure distilled water, the sections were incubated with antigen retrieval solution (sodium citrate buffer, pH 6) for 10 min at 90°C. Sections were washed in 1% PBS, treated with 1% glycine for 30 min, incubated with blocking solution (1% bovine serum albumin, 0.05% Tween 20, and 10% goat serum) at room temperature for 1 hour and then incubated with anti-Npt2a primary antibody (Slc34A1 antibody, Novus Biological, Centennial, CO) in a humidified dark chamber at 4°C overnight. Sections were washed and then incubated with polyclonal anti-rabbit biotinylated secondary antibody (VECTASTAIN Elite ABC Kit, Vector Laboratories, Burlingame, CA) diluted at 1:200 for 1.5 hours at room temperature in a dark chamber. Sections were washed and treated with 3% H_2_O_2_ at 37°C for 30 min then with avidin biotin solution for 60 min (VECTASTAIN Elite ABC Kit), washed in 3% PBS and tris-HCL (pH 7.6), and reacted with diaminobenzidine substrate (Sigma-Aldrich). For nuclear counterstaining, sections were stained with hematoxylin. Control incubations to assess nonspecific staining consisted of the same procedure except that the primary antibody was replaced by nonimmune serum.

#### Histological quantification

For histological quantification, the images were processed using Fiji (Fiji Is Just ImageJ) software 2.1.0/1.53c, version for Windows. Osteoid quantification was performed on the images of left tibia’s secondary spongiosa stained with Von Kossa. The ROI was focused on secondary spongiosa. All the ROIs were adjusted according the tibia size (described in the section of micro-CT analysis). Each ROI was split in square fields (from 7 to 12). The total bone area and the osteoid area were calculated in each square field, and the results were summarized. Then, the percentage of osteoid was calculated dividing the osteoid area to total bone area.

### Statistical analysis

Statistical analysis and graphs were performed with GraphPad Prism for Windows, version 7.0. Results were expressed as means ± SD for continuous variables. The comparison of continuous variables was performed using Student’s *t* test or one-way or two-way analysis of variance (ANOVA), as appropriate. *P* values of less than 0.05 were considered significant.

## Supplementary Materials

### This PDF file includes:

Figs. S1 to S4 Tables S1 and S2 Raw data

## Notes

View/request a protocol for this paper from *Bio-protocol*.
