# Supplementary material for: A novel therapeutic strategy for skeletal disorders: Proof of concept of gene therapy for X-linked hypophosphatemia
Source: Sci Adv. 2021 Oct 27;7(44):eabj5018. doi: 10.1126/sciadv.abj5018 (PMC8550245; doi:10.1126/sciadv.abj5018)
Supplement: Supplementary file 1 — Figs. S1 to S4 Tables S1 and S2 Raw data [file sciadv.abj5018_sm.pdf]

Science Advances
AAAS

Supplementary Materials for

A novel therapeutic strategy for skeletal disorders: Proof of concept of gene therapy for X-linked hypophosphatemia

Volha V. Zhukouskaya, Louisa Jauze, Séverine Charles, Christian Leborgne, Stéphane Hilliquin, Jérémy Sadoine, Lotfi Slimani, Brigitte Baroukh, Laetitia van Wittenberghe, Natalie Danièle, Fabienne Rajas, Agnès Linglart, Federico Mingozzi, Catherine Chaussain, Claire Bardet*, Giuseppe Ronzitti*

*Corresponding author. Email: gronzitti@genethon.fr (G.R.); claire.bardet@u-paris.fr (C.B.)

Published 27 October 2021, *Sci. Adv.* **7**, eabj5018 (2021)
DOI: 10.1126/sciadv.abj5018

This PDF file includes:

Figs. S1 to S4
Tables S1 and S2
Raw data

SUPPLEMENTARY FIGURE 1

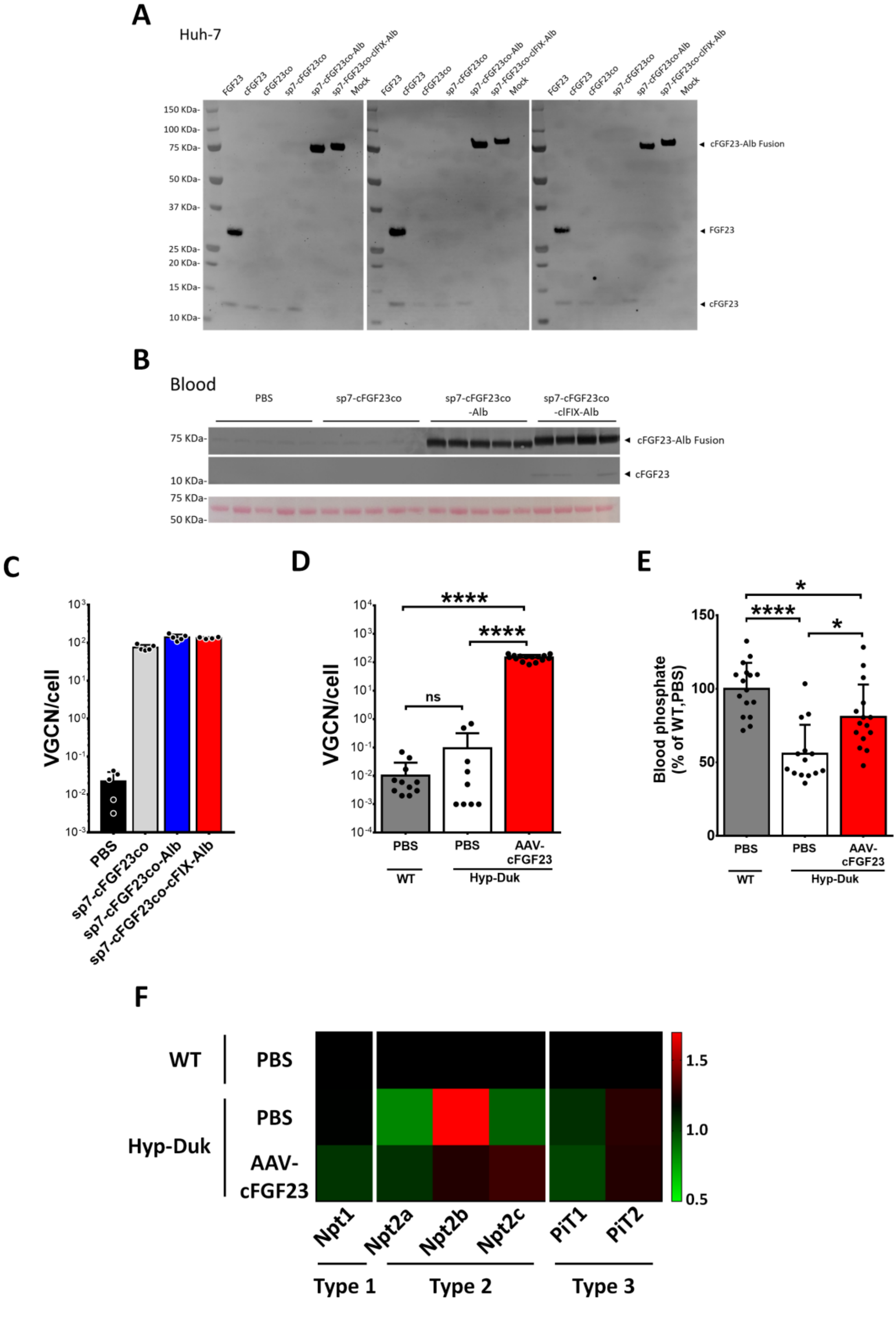

Figure S1. Engineering of the cFGF23 transgene for liver expression results in decreased FGF23 signaling in kidney in a mouse model of XLH. **A.** Western blot analysis of FGF23 isoforms expression in the medium of Huh-7 cells transfected as described in Figure 1. **B.** Western blot analysis of cFGF23 and cFGF23-Albumin fusion levels in blood of C57Bl6/J mice injected with AAV-cFGF23 as described in Figure 1. Ponceau staining of the membrane was used as loading control. **C.** Vector genome copy number per diploid cell in liver of C57Bl6/J mice. **D.** Vector genome copy number per diploid cell in liver of wild-type and Hyp-Duk mice treated with PBS or AAV-cFGF23. **E.** Blood phosphate levels measured one month after vector injection. **F.** Heatmap showing the expression of the indicated Na/Pi co-transporter mRNAs in kidney. Statistical analyses were performed by ANOVA (*$p<0.05$; ****$p < 0.0001$; ns: non-significant). All data are shown as mean ± SD (n=3 independent experiments in A; n=4-5 mice in B, C; n=14-16 mice from three independent experiments in D-F).

SUPPLEMENTARY FIGURE 2

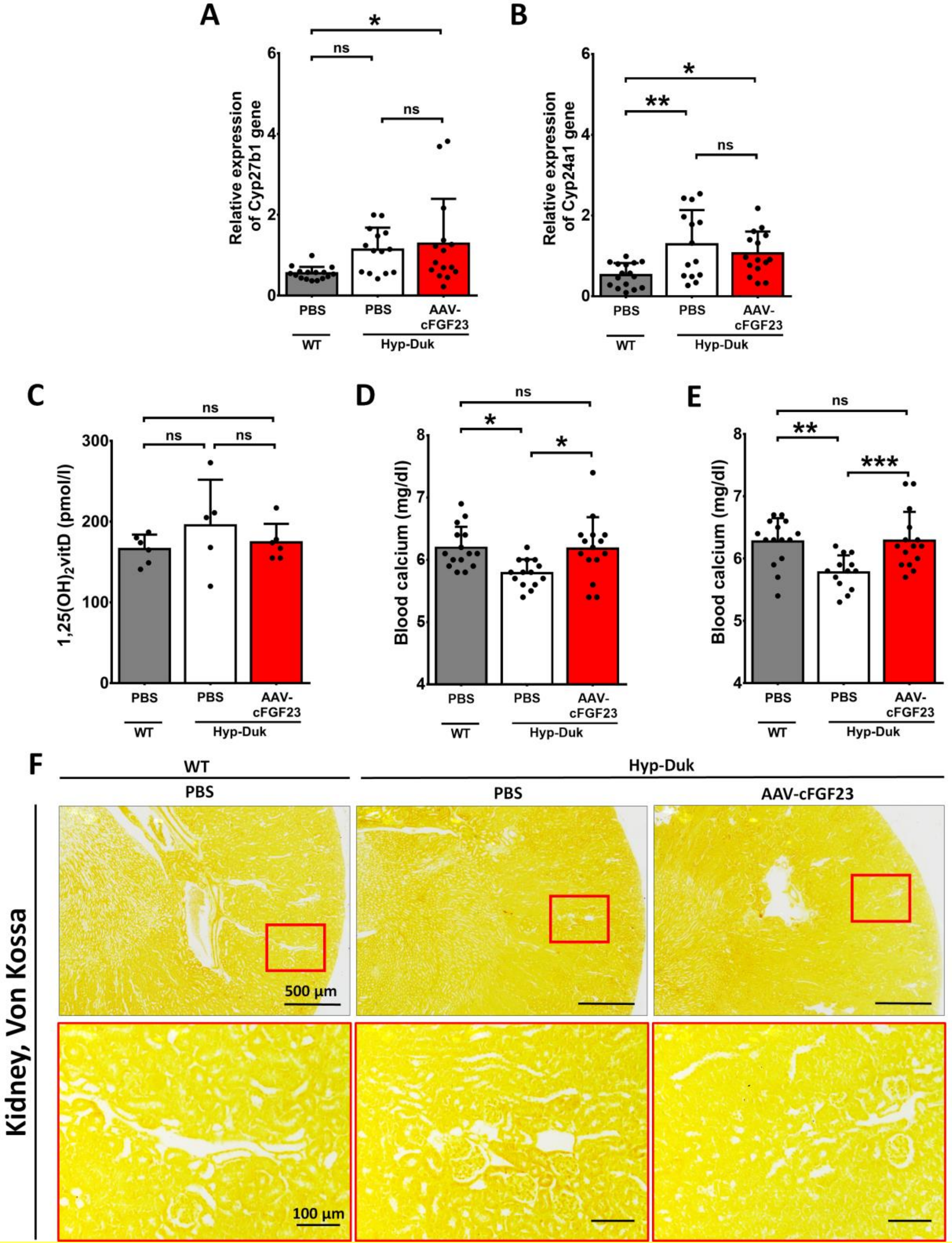

Figure S2. Evaluation of the off-target effects of AAV-cFGF23 treatment. Hyp-Duk mice were treated as described in Figure 1 and sacrificed three months after vector injection to evaluate the potential off-targets of the treatment. **A.** Expression levels of 25-hydroxyvitamin D-1 alpha hydroxylase (Cyp27b1) in kidney. **B.** Expression levels of 1,25-dihydroxyvitamin D(3) 24-hydroxylase (Cyp24a1) in kidney. **C.** Blood levels of 1,25$(OH)_2$-vitamin D. **D,E.** Blood calcium levels measured 1 month (**D**) and three months (**E**) after AAV-cFGF23 injection. **F.** Von Kossa staining of the kidney. Statistical analyses were performed by ANOVA (*$p < 0.05$; ** $p < 0.01$; *** $p < 0.001$; ns: non-significant). All data are shown as mean ± SD (n=14-16 mice per group from three independent experiments).

SUPPLEMENTARY FIGURE 3

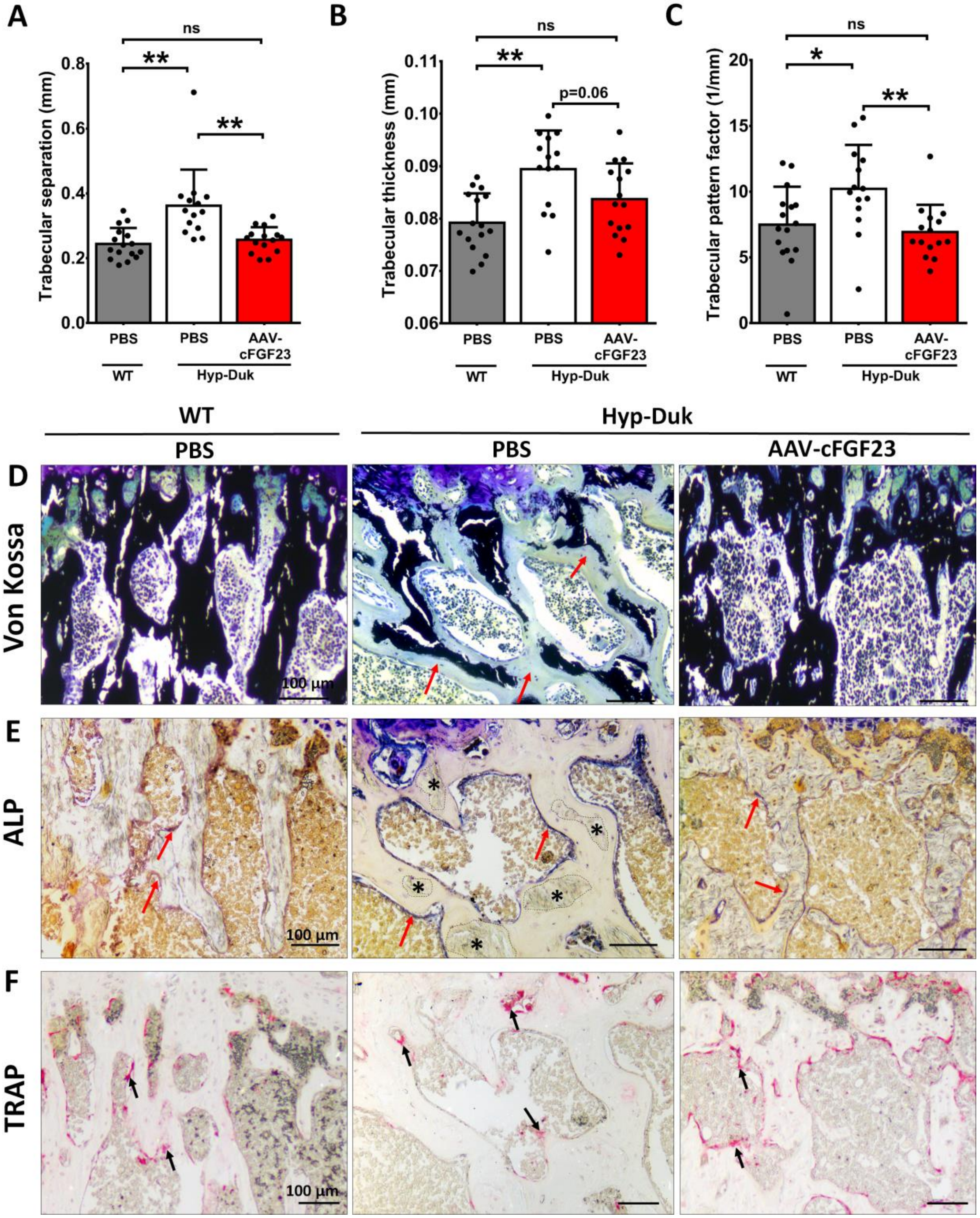

Figure S3. Impact of the AAV-cFGF23 on trabecular bone microarchitecture and on markers of bone metabolism. **A.** Trabecular separation, expressed in mm. **B.** Trabecular thickness, expressed

in mm. **C.** Trabecular pattern factor, expressed as connectedness of trabeculae per mm. **D.** Von Kossa staining. Red arrows indicate region of non-mineralized collagenous matrix i.e. the osteoid. **E.** ALP enzyme histochemistry performed on proximal tibia. Red arrows indicate ALP activity surrounding the region of osteoid accumulation. Mineralized bone is indicated with stars. **F.** TRAP enzyme histochemistry performed on proximal tibia. TRAP staining is indicated by red arrows. Statistical analyses were performed by ANOVA (*$p < 0.05$; ** $p < 0.01$; ns: non-significant). All data are shown as mean ± SD (n=14-16 mice per group from three independent experiments).

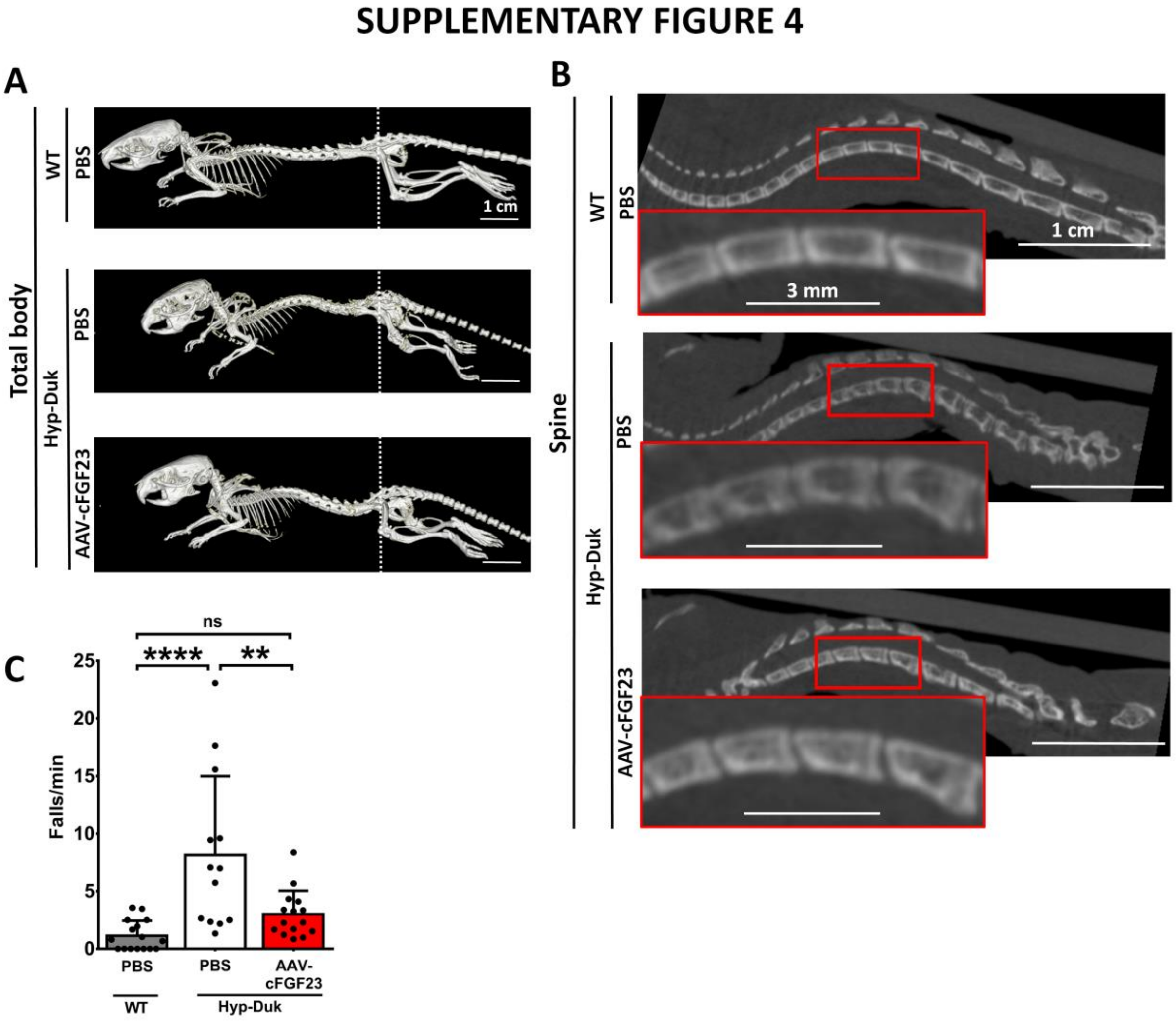

Figure S4. AAV-cFGF23 treatment rescue skeletal features and functional activity in Hyp-Duk mice. **A.** Representative images of total body scan by Micro-CT. **B.** Representative images of Micro-CT performed on spine. **C.** Four limbs hanging test performed three months after AAV-cFGF23 or PBS injection. Results are expressed as the number of falls per minute. Statistical analysis was performed by ANOVA (** $p < 0.01$; **** $p < 0.0001$; ns: non-significant). In C, data are shown as mean ± SD (n=14-16 mice per group from three independent experiments).

Table S1: Sacroiliac joint score for erosion in treated HypDuk AAV mice, untreated HypDuk and WT mice.

Mice #	#1	#2	#3	#4	#5	#6	#7	#8	#9	#10	#11	#12	#13	#14	#15	#16
**WT**	0	0	0	0	0	0	0	0	0	1	0	0	0	1	1	0
**HypDuk**	2.75	2.25	2	2.25	2.75	2.25	2.5	2.5	2.5	2	2	2.25	2.25	2.25	-	-
**HypDuk AAV**	0	0	0	1	0	1	0	1	0	1	0	0	0	0	1	-

Table S2: Scoring grid of erosions

**Variables**	**Score**	**Description**
Erosion of the sacroiliac joints	**0**	normal
	**1**	doubtful
	**2**	< 25% of the articular surface area affected
	**2.25**	≥ 25% to < 50% of the articular surface area affected
	**2.5**	≥ 50% to < 75% of the articular surface area affected
	**2.75**	≥ 75% to < 100% of the articular surface area affected
	**3**	all the articular surface area affected

RAW DATA

FIGURE 1C

	cFGF23co			FGF23			cFGF23-Alb fusion		
FGF23	521	678	389	4720	8870	3290			
cFGF23	365	141	243						
cFGF23co	112	168	81.4						
sp7-cFGF23co	511	229	279						
sp7-cFGF23co-Alb							38600	35700	14500
sp7-cFGF23co-clFIX-Alb							28000	8540	28500

FIGURE 1E

	cFGF23co				
sp7-cFGF23co-Alb					
sp7-cFGF23co-clFIX-Alb	489	484	62.8	701	

	cFGF23-Alb fusion				
sp7-cFGF23co-Alb	45700	47400	44100	32000	41700
sp7-cFGF23co-clFIX-Alb	73400	67200	98900	70900	

FIGURE 1F

PBS	sp7-cFGF23co	sp7-cFGF23co-Alb	sp7-cFGF23co-clFIX-Alb
5.3	4.7	5	7.2
4.7	5.4	5.2	6.5
4	4.6	6.2	6.2
4.6	4.8	4.9	5.4
5.8	4.8	4.2	

FIGURE 2B

WT	HypDuk	HypDukAAV
1.47	1.035	1.15
1.42	1.085	1.425
1.34	1.015	1.13
1.265	1.19	1.255
1.33	1.15	1.295
1.375	0.88	1.32
1.69	0.89	1.215
1.29	0.62	0.91
1.47	1.075	1.3
1.55	0.885	0.925
1.09	0.94	1.355
1.61	1.015	1.465
1.35	1.255	1.295
1.425	1.26	1.445
1.34		1.41
1.26		

FIGURE 2D

WT, PBS	HypDuk, PBS	HypDuk, AAV
104.7009	44.87179	61.96581
94.01709	49.1453	81.19658
126.0684	61.96581	81.19658
79.05983	57.69231	83.33333
96.15385	85.47009	70.51282
108.9286	44.64286	92.85714
121.4286	44.64286	96.42857
89.28571	46.42857	69.64286
80.35714	35.71429	51.78571
92.54499	67.86632	103.5714
92.54499	83.29049	53.98458
137.2751	57.06941	80.20566
63.23907	63.23907	35.47558
100.2571	60.15424	52.44216
114.1388		61.69666

FIGURE 3B

WT	HypDuk	HypDukAAV
15.20495	10.87655	19.56887
25.70033	15.97287	24.87082
20.01798	18.7977	34.08889
24.87811	14.79607	24.57849
28.80298	14.11313	16.75544
26.08814	20.53777	17.83621
25.85836	15.20323	22.29269
18.48401	17.8	20.25411
17.40003	20.92182	24.7931
28.73354	21.43279	20.54351
16.20819	28.07778	28.45993
28.30723	17.76464	21.79558
30.15606	14.74463	28.11837
24.91386	18.34078	20.41192
24.06729		22.78133
26.01409		

FIGURE 3C

WT	HypDuk	HypDukAAV
2.04106	1.3499	2.14339
3.34111	2.16959	2.72886
2.39832	2.01285	3.53226
2.93956	1.61267	3.13573
3.41729	1.52673	2.14289
3.03791	2.15382	2.34826
3.26905	1.83887	2.64072
2.64477	1.98408	2.63823
2.38805	2.17145	2.83075
3.77785	2.22553	2.81282
2.27444	2.81948	3.20316
3.27564	1.9803	2.63797
3.43019	1.82486	3.15987
3.16611	2.04828	2.58
3.11382		2.75379
3.3518		

FIGURE 3E

WT	HypDuk	HypDukAAV
57.32779	29.68763	42.72016
55.47149	30.90912	46.41427
54.72449	39.98334	45.11484
54.85197	36.53857	44.16394
51.11417	30.01028	44.15688
55.81922	38.5521	39.70242
56.21513	29.28501	49.00649
51.95526	31.56192	46.55362
58.11496	32.19372	45.05838
56.15469	40.76064	40.45907
53.2414	38.66259	47.6044
61.48581	40.13828	48.30287
66.60663	34.94652	48.31191
61.42648	40.33935	52.12733
65.66849		46.39366
60.7808		

FIGURE 3F

WT	HypDuk	HypDukAAV
0.21531	0.11207	0.18103
0.21124	0.12822	0.2205
0.22643	0.1382	0.15033
0.25134	0.14534	0.1872
0.23722	0.11658	0.15811
0.24793	0.16078	0.15751
0.24609	0.13184	0.21803
0.21337	0.13738	0.17834
0.24475	0.14217	0.21486
0.22081	0.17063	0.13173
0.22172	0.15968	0.1717
0.2564	0.16505	0.21177
0.30312	0.14425	0.20646
0.26593	0.14417	0.22
0.28904		0.23167
0.27959		

FIGURE 3H

WT	HypDuk	HypDuk AAV
12.84	30.64	6.79
12.63	46.41	5.71
11.5	35.24	10.01
12.51	59.22	12.62
11.15		
10.98		

FIGURE 3J

WT	HypDuk	HypDukAAV
66	217	89
64	177	107
66	228	80
55	189	68
68	227	105
49	406	164
73	284	82
43	280	80
63	320	63
57	334	109
34	227	87
63	305	100
49	272	113
51	231	99
		98

FIGURE 4B

WT	HypDuk	HypDukAAV
1.53	1.068	1.282
1.48	1.119	1.291
1.495	1.155	1.31
1.566	1.133	1.319
1.554	1.062	1.208
1.531	1.135	1.258
1.522	1.119	1.274
1.568	1.124	1.26
1.501	1.169	1.282
1.54	1.135	1.349
1.565	1.135	1.293
1.522	1.054	1.235
1.535	1.056	1.264
1.458	1.107	1.237
1.435		1.234
1.543		

FIGURE 4D

WT	HypDuk	HypDukAAV
1.789	1.166	1.4
1.733	1.264	1.38
1.809	1.239	1.38
1.759	1.29	1.458
1.787	1.263	1.486
1.769	1.338	1.432
1.701	1.291	1.366
1.75	1.255	1.425
1.659	1.19	1.368
1.65	1.195	1.376
1.733	1.345	
1.786	1.251	
1.678		
1.724		
1.752		
1.744		

FIGURE 4F

Time (months)	WT PBS															
0	14.6	12.6	16.5	17	15.7	17.5	16.4	19.6	17.9	20.3	18.4	15.8	15.7	15.4	15.4	17.7
1	24.3	22.6	23.1	27.2	27.3	27.8	24.7	27.3	25.9	28.1	27.6	25.1	24.6	24.3	22.7	25.1
2	27.3	25.1	25.3	30	30.2	33.6	29.6	30.7	27	31.2	32.2	26.9	27.3	26.2	24.4	29.3
3	28.3	27.2	26	30.5	32	36.5	32.1	32.3	30.4	34.2	33.2	30.4	30.1	28.4	26.6	31.2

Time (months)	HypDuk, PBS													
0	11.2	12.4	13.5	13.4	9.5	12.3	13.2	16.5	14.8	12.2	13.6	14.7	12.3	12.2
1	16.8	19	20.4	19.8	16.1	18.2	18.5	21	21.5	20.7	20.5	19.7	17.7	17.3
2	18.2	21.7	21.4	21.4	18.5	19.8	19.8	20.9	22.4	22.2	22.2	22.4	19.3	18.6
3	18.3	21.5	22.5	21.9	19.2	22	19.5	22.4	23.5	23.6	23.8	22.4	20.2	20.5

Time (months)	HypDuk, AAV														
0	11.4	12.4	13.4	12.5	13.5	12.9	12.9	11.5	14.8	16.5	12.7	12.7	11.2	14.5	14.2
1	19.6	21.1	22.7	20.4	21.9	20.5	19.4	19.5	23	23.9	19.1	19.9	20.3	21.5	21.5
2	21.5	22.7	24.7	22.3	18.3	23.4	21.3	22.5	24.4	27.4	21	21.9	23.6	24.3	23.3
3	22.3	23.7	25.7	22.5	25	25.2	22.1	23.1	26.3	27.4	23.4	24.1	24.7	24.1	24.6

FIGURE 4G

Time (months)	WT PBS															
0	72.2	68.1	74.8	74.5	71.9	79.8	77.7	79.9	79	80.3	80.2	78.6	79.1	71.7	76	75.3
1	87.7	85.3	81	90.2	87.4	93.8	89.2	87.1	89.2	92.4	91.5	89.6	85.3	88.4	82.4	86.7
2	86.9	85.6	88.3	90.5	89.7	92.7	89.8	95.6	94.4	93	95.1	90.9	88.9	91.6	87.1	91.6
3	89.5	87.4	93.5	90.7	90.1	95	95	94.4	94.8	97	96.3	91.2	91.3	93.6	89.1	92

Time (months)	HypDuk, PBS													
0	63.2	66.7	67.5	68	65.8	70.8	70.5	71.5	68.2	71.2	72.9	74.2	71.2	70.8
1	74.6	77.1	77.3	79.3	73	76	74.5	82.3	80.8	81.2	81.4	78.7	73.7	73.6
2	75.1	79.9	80.7	78.3	74.5	77.5	76.8	80.9	83	82	82.6	82	75.2	77.6
3	77.5	82.2	83.2	83	73.5	78.8	83.4	81.5	86.7	83.3	85.2	83.3	80.9	79.9

Time (months)	HypDuk, AAV														
0	66.6	68.2	67.2	66.1	67	72.7	72.6	64.5	68.9	72.5	70.4	71.6	64.6	72.1	71.2
1	75.8	77.9	79.9	77.2	76.9	79.6	78.5	76.7	79.8	83.5	77.2	79.3	80.3	82.1	80
2	76.3	79.6	84.2	81.2	82.2	84.6	81.5	83.8	87	86.9	79.3	84.3	85.5	85.1	81.9
3	80.6	83.2	86.9	82.2	86.3	84.8	81.6	85	87.3	86.7	84.2	85.3	86.6	85.6	85

FIGURE 4H

Time (months)	WT PBS															
0	64.2	61	66.9	70.8	67.6	66.4	68.5	71.7	64.9	70.2	66.5	72.4	69.7	65.2	64.1	72.3
1	79.9	77	78.6	82.1	82.4	80.4	79.6	80.4	77.6	80.1	80.8	84.1	84.2	79	76.6	83.8
2	81.4	79.6	80.7	83.4	83.6	85.2	81.4	85	80	81.5	83.3	85.6	86.5	82.4	78.4	86.8
3	79.2	82.7	82	84.5	83.5	85.8	84.7	86	83.5	82.2	86.5	87.8	88	84.2	81.7	87

Time (months)	HypDuk, PBS													
0	49.3	52.3	55	52.8	46.1	52.2	52.6	53.6	50.5	53.5	51.2	52.1	47.9	46.7
1	53	58.3	59.8	58.7	47.9	58.9	55.3	57.8	54.9	59.8	59.3	57.6	53.4	51.4
2	54.6	61	63.2	61.4	50.3	59.9	56.9	60.3	58.7	60.7	61	59.9	53.7	51.9
3	53.1	61.1	62	60.6	53.2	62.8	57.9	61.3	59	60.8	62.7	59.3	55.2	53.9

Time (months)	HypDuk, AAV														
0	48.6	54.3	53.3	55.1	55.2	51.4	51.3	48	49.7	61.5	54.2	52.9	47.5	53	53.5
1	61.8	65.8	62.7	65.1	62.1	58.6	60.3	60.4	60.8	67.2	62.8	61.6	62.3	61.4	60.4
2	63.2	69.8	66.5	67.8	64.2	61.2	60.7	64.1	62.5	67.5	64.5	65.8	65.2	64.6	64
3	64	72.4	65.6	67.7	63.4	63.8	63.1	67.9	64.8	72.3	65.7	67.1	68.6	65.2	64

FIGURE 4J

WT	HypDuk	HypDukAAV
0	2.75	0
0	2.25	0
0	2	0
0	2.25	1
0	2.75	0
0	2.25	1
0	2.5	0
0	2.5	1
0	2.5	0
1	2	1
0	2	0
0	2.25	0
0	2.25	0
1	2.25	0
1		1
0		

SUPPLEMENTARY FIGURE 1C

PBS	sp7-cFGF23co	sp7-cFGF23co-Alb	sp7-cFGF23co-cFIX-Alb
0.04214	67.16	174.91	138.44
0.03392	93.3	107.75	130.16
0.00726	82.48	151.04	140.97
0.00319	62.47	124.4	124.94
0.02481	66.49	137.15	

SUPPLEMENTARY FIGURE 1D

WT	KO	KO, AAV
0.069	0.484	149.814
0.003	0.032	170.843
0.003	0.685	195.453
0.004	0.005	153.774
0.006	0.001	197.758
0.017	0.014	120.029
0	0.001	162.464
0.006	0.001	133.768
0.002	0.001	156.34
0.007	0	137.092
0.043	0	168.702
0	0	155.448
0	0	95.153
0.002	0	82.535
0		101.652
0		

SUPPLEMENTARY FIGURE 1E

WT, PBS	HypDuk, PE	HypDuk, AAV
105.3719	45.45455	66.1157
109.5041	51.65289	70.24793
99.17355	55.78512	84.71074
90.90909	55.78512	80.57851
109.6552	82.75862	80.57851
132.4138	80.68966	107.5862
80.68966	41.37931	115.8621
74.48276	103.4483	76.55172
80.68966	57.93103	70.34483
122.069	42.66212	128.2759
71.67235	40.95563	47.78157
90.44369	42.66212	58.02048
109.215	35.83618	90.44369
117.7474	44.3686	59.72696
110.9215		75.08532

SUPPLEMENTARY FIGURE 1F

	WT	KO	AAV-cFGF23
Npt1	1	0.99537	0.896359
Npt2a	1	0.73343	0.904811
Npt2b	1	1.697005	1.092473
Npt2c	1	0.809069	1.165583
PiT1	1	0.907158	0.865354
Pit2	1	1.113163	1.09296

SUPPLEMENTARY FIGURE 2A

WT	HypDuk	HypDukAAV
0.7	1.995	0.69
0.57	0.575	1.37
0.99	1.98	3.82
0.37	1.655	1.22
0.365	1.475	1.115
0.735	0.545	1.265
0.575	1.24	0.7
0.435	1.11	0.22
0.595	1.09	0.815
0.505	1.145	3.69
0.575	0.585	0.59
0.42	0.415	0.635
0.47	1.55	0.45
0.545	0.545	2.165
0.405		0.49
0.53		

SUPPLEMENTARY FIGURE 2B

WT	HypDuk	HypDukAAV
0.09	0.53	1.7
0.57	2.43	0.97
0.28	0.34	0.47
0.83	2.4	0.33
0.99	2.54	0.77
0.57	1.97	1.32
0.86	0.86	1.61
0.3	0.49	2.18
0.8	1.83	0.69
0.85	0.27	0.32
0.21	0.78	0.9
0.49	1.32	1.51
0.46	0.51	0.84
0.78	1.78	1.4
0.16		0.91
0.19		

SUPPLEMENTARY FIGURE 2C

WT	HypDuk	HypDukAAV
149	120	155
165	168	155
174	273	174
187	211	217
180	205	167
141		178

SUPPLEMENTARY FIGURE 2D

WTPBS	KOPBS	KOAAV
5.9	5.8	6
5.9	5.4	5.4
5.8	5.8	6.1
6.4	5.5	6.4
6	5.7	5.4
6.2	5.9	6
6.4	5.6	6.3
6.1	5.8	6.4
6.1	5.6	6.3
6	6	6.4
6.6	6.2	5.6
5.8	6	6.7
6.1	5.7	6.2
6.7	6	6.1
6.9		7.4

SUPPLEMENTARY FIGURE 2E

WTPBS	KOPBS	KOAAV
6.6	5.3	6.3
6.4	5.6	6
5.7	5.4	6.5
5.4	5.8	5.7
6.3	6.1	5.9
6.7	6.1	7.2
6.5	5.8	6.8
6.4	6.2	7.2
6.7	5.7	6.2
6.3	5.8	6.2
6	5.9	5.8
6.3	5.9	6.2
6.3	5.5	6.3
6.6		5.9
5.9		6.1

SUPPLEMENTARY FIGURE 3A

WT	HypDuk	HypDukAAV
0.30869	0.37809	0.32909
0.19653	0.25788	0.24784
0.34621	0.29263	0.19474
0.2575	0.38915	0.21351
0.18786	0.71179	0.30625
0.26942	0.33255	0.30285
0.241	0.36823	0.27377
0.24433	0.34689	0.27151
0.31592	0.34284	0.24041
0.17843	0.26119	0.26394
0.2808	0.28026	0.1955
0.21919	0.30904	0.26827
0.21399	0.3913	0.22129
0.21855	0.40076	0.25545
0.22566		0.26261
0.20281		

SUPPLEMENTARY FIGURE 3B

WT	HypDuk	HypDukAAV
0.0745	0.08057	0.0913
0.07692	0.07362	0.09114
0.08347	0.09339	0.09651
0.08463	0.09175	0.07838
0.08429	0.09244	0.07819
0.08588	0.09536	0.07595
0.0791	0.08268	0.08442
0.06989	0.08971	0.07677
0.07286	0.09635	0.08758
0.07606	0.0963	0.07304
0.07126	0.09959	0.08885
0.08642	0.08971	0.08262
0.08791	0.0808	0.08899
0.07869	0.08954	0.07912
0.07729		0.08273
0.07761		

SUPPLEMENTARY FIGURE 3C

WT	HypDuk	HypDukAAV
7.58902	15.61901	12.67681
6.14817	15.09153	7.99347
0.68645	10.22982	7.71213
7.07605	9.3983	6.15609
8.96958	9.9469	6.09853
5.52104	7.86394	8.32762
5.5731	11.64459	4.85725
11.96242	8.72884	6.2055
10.51028	6.74009	6.19053
7.17753	10.32536	7.04753
12.17111	2.57879	5.77918
5.38398	12.84103	7.27537
4.74965	12.38515	5.00578
8.22518	9.472	8.55285
9.04576		3.94511
8.83368		

SUPPLEMENTARY FIGURE 4C

WT	HypDuk	HypDuk, AAV
3.48	23.08	1.72
1.03	7.06	2.29
1.95	5.73	3.35
2.5	15.58	0.98
3.57	17.65	0.84
0	1.33	5.67
0	9.45	1.67
1.67	2.64	4.33
0.8	9.6	8.39
2.5	6.98	4.11
0	2.35	3.27
0	2.18	1.53
0	2.5	2.26
0		3.39
0		1.22
0.67		
